# Limited Joint Mobility in Type 1 Diabetes: Diabetic Cheiroarthropathy, a Neglected Entity

**DOI:** 10.1210/jcemcr/luad068

**Published:** 2023-07-13

**Authors:** Jayshree Swain, Brij Teli, Abhay Sahoo, Lavanya Kasukurti

**Affiliations:** Department of Endocrinology, IMS & Sum Hospital, Bhubaneswar, 751003, Odisha, India; Department of Endocrinology, IMS & Sum Hospital, Bhubaneswar, 751003, Odisha, India; Department of Endocrinology, IMS & Sum Hospital, Bhubaneswar, 751003, Odisha, India; Department of Endocrinology, IMS & Sum Hospital, Bhubaneswar, 751003, Odisha, India

**Keywords:** limited joint mobility, diabetic cheiroarthropathy, Namaste sign, Table Top sign

## Abstract

Musculoskeletal disorders are common in type 1 and type 2 diabetes mellitus. Among them, diabetic cheiroarthropathy (DCA), more commonly seen in type 1 diabetes, is a late complication that often causes physical and emotional disturbance. DCA, characterized by movement restrictions in the small joints of hands, is usually a clinical diagnosis and bears significance owing to the functional hand disabilities that it causes and its association with various microvascular complications, most importantly retinopathy. A 24-year-old male patient, with type 1 diabetes of 20 years duration, presented to us with difficulties in performing fine motor tasks such as buttoning his shirt and with positive “Namaste” sign and “Table Top” sign. He had reduced sensation on monofilament testing, decreased vibration perception threshold, and a nerve conduction study suggested distal sensory demyelinating and axonal polyneuropathy. He had a restrictive pattern on pulmonary function tests, normal lung parenchyma on high-resolution computed tomography of his thorax, proliferative diabetic retinopathy, proteinuria, vitamin D deficiency, and subclinical hypothyroidism. He was followed closely with tight glycemic control and physiotherapy. In rural setups, DCA can act as a mirror to screen for macrovascular and microvascular complications if not already done routinely or previously. Management includes physiotherapy, glycemic control, patient education, and regular follow-up, with surgical procedures being only the last option.

## Introduction

Diabetic cheiroarthropathy (DCA) is a musculoskeletal condition seen in patients with diabetes, more commonly in type 1 diabetes. Other musculoskeletal conditions affecting diabetic patients involving upper or lower limbs include adhesive capsulitis, diabetic sclerodactyly, Dupuytren contracture, carpal tunnel syndrome, plantar fasciitis, and flexor tenosynovitis as well as other tendinopathies [1]. DCA is characterized by limitation in joint movement, significantly in the small joints of hands. Skin thickening and waxiness are also common, particularly on the dorsal surface of the fingers. Cheiroarthropathy (Greek word for hand *cheiros*) may alternatively be called limited joint mobility, diabetic stiff hand syndrome, and limited joint mobility with sclerodactyly. Nowadays, the term *limited joint mobility* is used more commonly, but we prefer *cheiroarthropathy* in this discussion, as it is unequivocal. Although simple to diagnose, it is often a neglected finding. If picked up during outpatient clinical examination, it can act as a useful aid to offer immediate screening to the patient for microvascular and macrovascular complications of diabetes.

## Case Presentation

**Figure 1.**
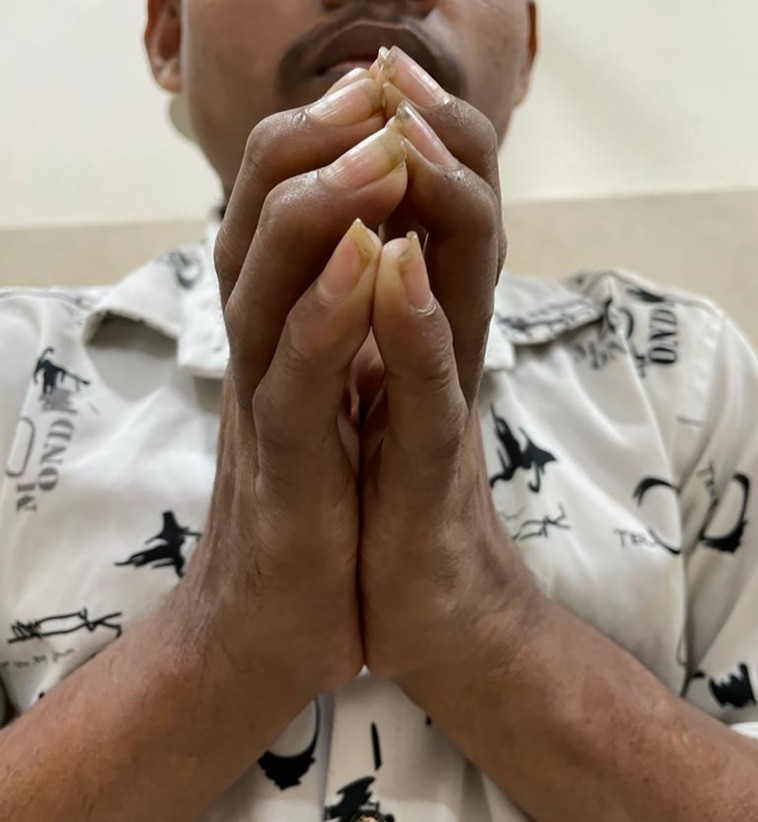
The “Namaste” sign is a classical bedside sign to demonstrate diabetic cheiroarthropathy, in which the patient is unable to oppose both hands closely.

**Figure 2.**
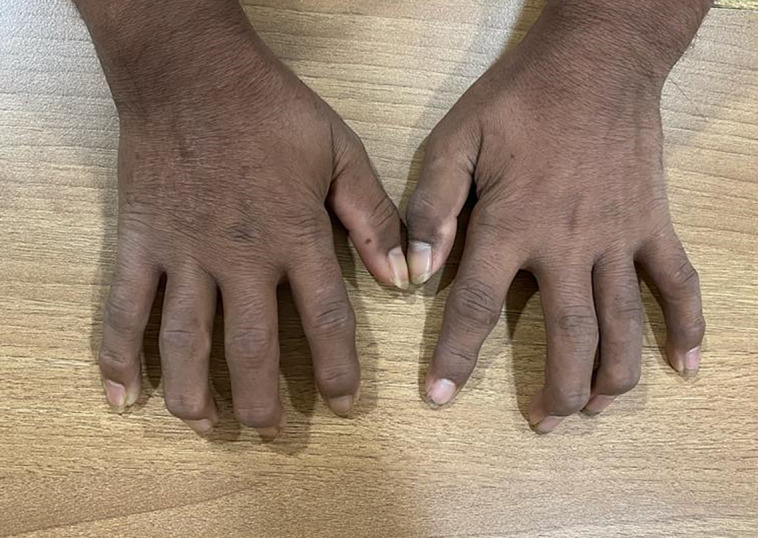
“Table Top” sign demonstrating the stiffness of fingers, in which the patient cannot lay hands flat on the table.

**Figure 3.**
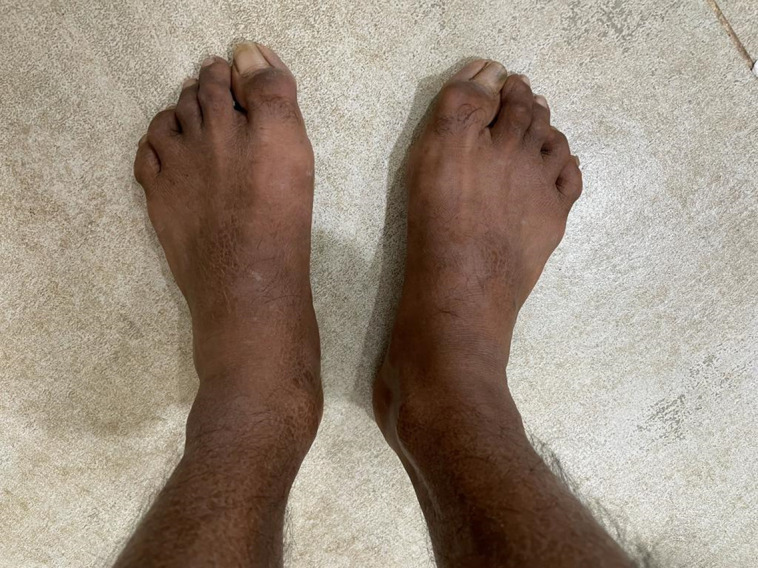
Hallux valgus deformity due to foot involvement in diabetic cheiroarthropathy.

We present case of a 24-year-old male patient, with type 1 diabetes of 20 years duration, normotensive, on thrice daily insulin regimen with poor compliance and poor glycemic control, and without proper monitoring or follow-up to any physician. He presented to us with a complaint of stiffness in both hands, which was progressive over last 5 years, painless, and associated with difficulty in performing his daily activities like buttoning shirts, writing, and even self-administrating insulin. He had fixed flexion deformity at bilateral proximal interphalangeal (PIP) joints, associated with skin tightening and cord-like induration in the palms at the metatarsophalangeal joint level. He had no history of hand trauma, thickened nerves, hypopigmented patches, pain, nodule in the joints, or Raynaud phenomenon. The “Namaste” or “Prayer” sign was demonstrable on joining hands opposed to each other (Fig. 1) and so was the “Table Top” sign (Fig. 2). Tinel's sign and Phalen's sign were negative, ruling out carpal tunnel syndrome. There was no evidence of adhesive capsulitis of the shoulder, trigger finger, or Dupuytren contracture. However, there was atrophy of thenar eminence. He had a foot at risk with bilateral hallux valgus but without any signs of active ulceration (Fig 3). There was no arch-related foot abnormality or Charcot arthropathy. The thyroid gland was normally palpable. Sexual maturity rating was A1, P5, testicular volume bilaterally 10 to 12 cc, and stretched penile length of 10 cm.

## Diagnostic Assessment

**Figure 4.**
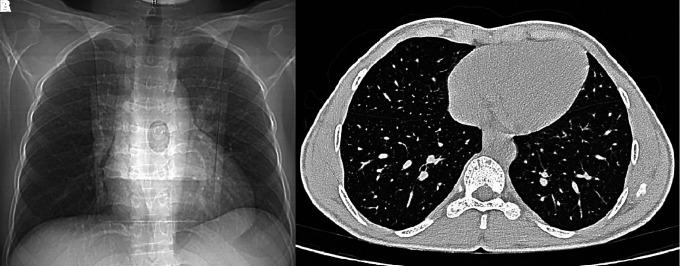
X-ray chest posteroanterior view (A) showing mildly prominent bronchovascular markings and crowding of ribs and HRCT thorax (B) axial plane showing normal lung parenchyma and mild flattening of anterior chest wall. The restrictive pattern on spirometry and DLCO when corroborated with chest imaging indicate the cause of restriction to be extraparenchymal, due to chest wall deformity in diabetic cheiroarthropathy.

**Figure 5.**
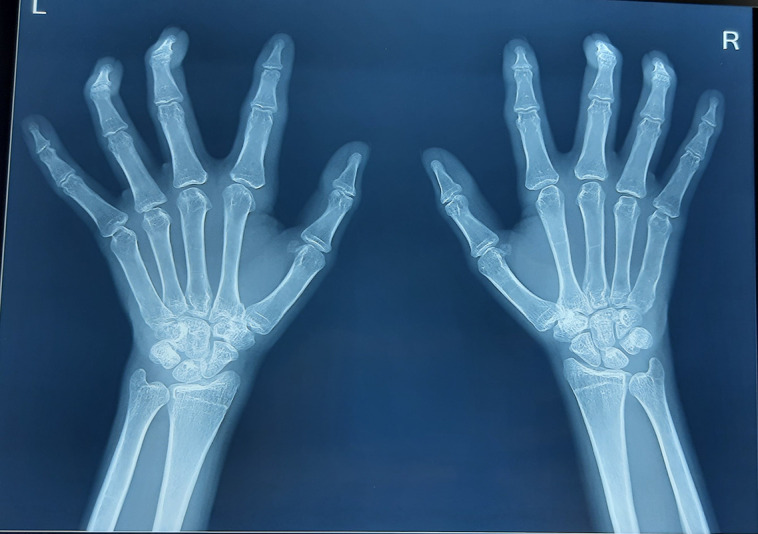
X-ray bilateral hand anteroposterior view demonstrating the fixed flexion deformity in hands in case of diabetic cheiroarthropathy.

The neuropathic assessment suggested decreased monofilament score (3/6 bilateral) and diminished hot and cold perception in the bilateral foot. The vibration perception threshold was decreased bilaterally in the feet (40 mV on the left side, 38 mV on the right side). The nerve conduction study suggested demyelinating and axonal polyneuropathy (prolonged onset latency and normal conduction velocity in bilateral median nerves and low amplitude in bilateral peroneal and tibial nerves with nonrecordable tracing from bilateral sensory median and sural nerves) in all 4 limbs. Fundoscopy was suggestive of proliferative diabetic retinopathy. Pulmonary function tests revealed a moderate restrictive pattern on spirometry (reduced total lung capacity, reduced FEV1 at 60%predicted [pred] and FVC 60%pred, with normal FEV1/FVC) and carbon monoxide transfer factor suggested extra parenchymal restriction (normal diffusing capacity of the lungs for carbon monoxide [DLCO] 82%pred, reduced alveolar volume V_A_ 62%pred and increased transfer coefficient kCO 156%pred). Chest x-ray and high-resolution computed tomography (HRCT) of his thorax revealed normal lung parenchyma consistent with extra parenchymal restrictive pattern on spirometry and DLCO (Fig. 4). Hand radiographs were consistent with fixed flexion deformity at bilateral distal interphalangeal (DIP) joints, without any erosions or inflammatory or neuropathic arthropathy-related changes (Fig. 5). Ultrasound of the hand suggested thickened flexor tendons, while that of abdomen was normal. Laboratory investigations were as mentioned in Table 1. The biochemical parameters suggested proteinuria, vitamin D deficiency, and subclinical hypothyroidism (anti-TPO negative). The workup was negative for any rheumatological or inflammatory markers (RA factor, C-reactive protein, creatine phosphokinase, anti-nuclear antibody) and also for celiac disease (anti-tissue transglutaminase antibody).

**Table 1.** Laboratory investigations

Investigation	Value Conventional unit (SI unit)	Reference range Conventional unit (SI unit)
Hemoglobin	12.9 g% (129 g/L)	14-18 g% (140-180 g/L)
Total WBC	9400/mm^3^ (9.4 × 10^9^/L)	4000-10000/mm^3^ (4-10 × 10^9^/L)
Platelets	2.5 lac/mm^3^ (250 × 10^9^/L)	1.5-3 lac/mm^3^ (150-300 × 10^9^/L)
MCV	73.6 µm^3^ or fL	75-96 µm^3^ or fL
ESR	10/first hour	5-15/first hour
Urea	28 mg/dL (10 mmol/L)	6-40 (2-14 mmol/L)
Creatinine	0.8 mg/dL (70.72 µmol/L)	0.6-1.5 (53-132 µmol/L)
Sodium	136 meq/L or mmol/L	135-145 meq/L or mmol/L
Potassium	4.4 meq/L or 4.4 mmol/L	3.5-5.5 meq/L or mmol/L
Calcium	9.2 mg/dL (2.3 mmol/L)	9-11 mg/dL (2.1-2.75 mmol/L)
Phosphorus	4.7 mg/dL (1.52 mmol/L)	2.5-5 mg/dL (0.8-1.6 mmol/L)
25-OH vitamin D	14.03 ng/mL (35.02 nmol/L)	>30 ng/mL (>74.88 nmol/L)
Bilirubin (direct)	0.1 mg/dL (1.71 µmol/L)	0-0.2 mg/dL (0-0.34 µmol/L)
Bilirubin (total)	0.3 mg/dL (5.13 µmol/L)	0.2-1 mg/dL (0.34-1.71 µmol/L)
SGOT (AST)	22 IU/L (0.37 µkat/L)	5-40 IU/L (0.08-0.67 µkat/L)
SGPT (ALT)	23 IU/L (0.38 µkat/L)	5-40 IU/L (0.08-0.67 µkat/L)
ALP	179 IU/L (2.99 µkat/L)	42-119 IU/L (0.70-1.99 µkat/L)
Serum protein	6.9 g/dL (69 g/L)	6.3-8.4 g/dL (63-84 g/L)
Serum albumin	3.8 g/dL (38 g/L)	3.5-5 g/dL (35-50 g/L)
Serum globulin	3.1 g/dL (31 g/L)	2.3-3.6 g/dL (23-36 g/L)
Urine routine & microscopy	Glucose 4+, Protein 2+, Ketones- Negative	Negative
Urine albumin	52.35 mg/dL	0.3-21 mg/dL
Urine creatinine	129.12 mg/dL	39-259 mg/dL
UACR	405.43 mg/g	<30 mg/g
FPG	252 mg/dL (13.99 mmol/L)	80–130 mg/dL (4.4-7.2 mmol/L)
2 hour PPG	332 mg/dL (18.426 mmol/L)	<180 mg/dL (<10 mmol/L)
HbA1c	11.8% (105 mmol/mol)	<7% (53 mmol/mol)
T3	103.25 ng/dL (1.59 nmol/L)	84.42-201.30 ng/dL (1.3-3.1 nmol/L)
T4	9.18 µg/dL (118.1 nmol/L)	5.13-14.06 µg/dL (66-181 nmol/L)
TSH	5.98 µIU/mL or mIU/L	0.27-4.2 µIU/mL or mIU/L
Anti-TPO	1.9 IU/mL	<9 IU/mL
RA factor	2.3 IU/mL	<30 IU/mL
CRP	1.6 mg/dL	<8 mg/dL
CPK	198 IU/L (3.31 µkat/L)	25-200 IU/L (0.42-3.34 µkat/L)
tTG IgA antibody	1.11 IU/mL	<4 IU/mL
ANA	Negative (< 1:40)	< 1:40

Abbreviations: ALP, alkaline phosphatase; ALT, alanine aminotransferase; ANA, anti-nuclear antibody; anti-TPO, anti–thyroid peroxidase; AST, aspartate aminotransferase; CPK, creatine phosphokinase; CRP, C-reactive protein; ESR, erythrocyte sedimentation rate; FPG, fasting plasma glucose; MCV, mean corpuscular volume; PPG, postprandial glucose; SGOT, serum glutamic-oxaloacetic transaminase; SGPT, serum glutamic-pyruvic-transaminase; TSH, thyrotropin (thyroid-stimulating hormone); tTG IgA, tissue transglutaminase IgA; UACR, urinary albumin creatinine ratio; WBC, white blood cells.

## Treatment

We started the patient with multiple daily injection regimen for insulin and followed him up stringently. He underwent one session of pan retinal photocoagulation. Low-dose angiotensin receptor blockers, vitamin D, and levothyroxine supplementation were started. The patient was advised regular stretching exercises, physiotherapy, and a brace for hand deformity. He was also given customized footwear and foot care education.

## Outcome and Follow-up

At the 6-month follow-up assessment, the patient showed improvement in glycemic control (HbA1c 7.5% or 58 mmol/mol) as well as normalization in thyroid parameters. He had significant improvement in generalized well-being, with modest symptomatic improvement in finger mobility. We plan to further offer him corrective surgery for his hand deformity.

## Discussion

Musculoskeletal disorders are associated with both type 1 and type 2 diabetes. Among these conditions, DCA bears significant clinical importance. DCA can develop in both type 1 and type 2 diabetes, but it is seen more commonly in type 1 diabetes and has a male predominance with a median duration of 10 years. It has a prevalence of 30% in type 1 diabetes and about 18% in type 2 diabetes [2, 3]. Characteristics of DCA include movement restriction at the small joints of hands, namely the metacarpophalangeal, proximal interphalangeal, and distal interphalangeal joints. Its usual onset is from the ulnar side of the hand, spreading radially later. DCA can also affect feet, characterized by impaired toe and foot joint mobility, thickening, and contracture of skin over the toes and plantar fascia. Although the literature has described the involvement of both the upper and lower limbs in DCA, our case had predominant involvement of the distal upper limb rather than the lower limb, and not of any other joints. The “Prayer” or “Namaste” sign is the classical finding in patients with DCA, demonstrated by failure to fully extend fingers of both hands and inability to approximate one or more fingers of both hands by opposition of palmar surfaces of proximal and distal interphalangeal joints with palms pressed together and fingers fanned. The Table Top sign is demonstrated by asking the patient to touch the table with the palmar surfaces of both hands and if the patient cannot completely keep digits or palms flat on the table, it is considered to be positive. Our case showed these classical signs along with scleroderma-like skin thickening. However, younger age of onset and absence of pain, Raynaud phenomenon, or other systemic manifestations like digital ulcers or gastrointestinal symptoms, negative anti-nuclear antibody, absence of features of arthritis on x-ray, with normal lung parenchyma on HRCT thorax, and most importantly the temporal correlation of clinical features with the duration of diabetes attributes the findings in our case to DCA more than systemic sclerosis, seronegative arthritis, or any other rheumatological or systemic condition.

Rosenbloom et al first demonstrated that there might be a strong association between the severity of joint limitation and the prevalence of microvascular disease in type 1 diabetes, most significantly with diabetic retinopathy followed by nephropathy and neuropathy [2]. They reported a 3-fold increased risk of clinically apparent microvascular disease in patients with DCA. Other studies also have individually demonstrated this finding, making the presence of DCA a significant indicator of microvascular complications [4]. Our patient had proliferative diabetic retinopathy, nephropathy, and neuropathy at the time of presentation with DCA. Very few studies have described the association of DCA with macrovascular complications such as hypertension and atherosclerosis. Arkkila et al found that 33% of diabetic patients with DCA had hypertension vs 14% of patients without DCA [5]. However, at 2 years of follow-up, they recorded no significant association between DCA and hypertension. Our patient did not develop hypertension despite having nephropathy, until the last follow-up visit.

The pathogenesis of DCA is multifactorial. Advanced glycation end-product (AGE) formation due to chronic hyperglycemia and collagen cross-link formation with the extensive proliferation of collagen in subcutaneous tissues, muscle, skin, and periarticular tissues are suggested to be the driving processes. Also, microangiopathy of blood vessels of the skin and subcutaneous tissues, causing reduced blood supply and thereby low-grade chronic ischemia causing skin fibrosis may give the appearance of tight, waxy skin [4, 6, 7]. Buckingham et al in 1984 studied pulmonary function in 3 diabetic patients with limited joint mobility, which showed a restrictive pattern in all 3 patients, suggested by reduced vital capacities and total lung capacities. It was suggested that restrictive pulmonary disease may be due to changes in chest wall compliance or changes in lung tissue resilience [7]. Our patient also had similar findings on spirometry with a restrictive pattern, which when further evaluated by HRCT thorax and DLCO, revealed it to be due to extra parenchymal restriction, which in this case can be attributed to the reduced chest wall compliance associated with DCA, with essentially normal lung parenchyma. Diagnosis of DCA is more often made clinically. However, modalities like ultrasound may show thickening in flexor tendon sheaths and subcutaneous tissues. Magnetic resonance imaging may show thickening and edema of flexor digitorum tendons and tendon sheath enhancement, without involvement of extensor tendons [8]. However, imaging modalities hardly have any consequence on prognosis and management. Treatment for DCA involves strict glycemic control. Early glycosylation of skin collagen can be decreased by improved glycemic status [9]. However, the long-term, cumulative damage due to the binding of AGEs to collagen is unlikely to be reversed. Physiotherapy plays a vital role in increasing hand mobility. Various studies have evaluated targeted therapy for AGE accumulation and production, but still there is no recommendation for their clinical use due to lack of evidence and safety concerns. These include alagebrium chloride, aminoguanidine, vitamin B products (thiamine, pyridoxamine, benfotiamine), oral antidiabetics (rosiglitazone, metformin, sulfonylureas), angiotensin receptor blockers, and AGE-restricted diets [10]. Surgical correction is usually the last option after exhaustive conservative management and it includes excision of fibrotic adhesions, nodules, and degenerated areas, and rarely includes reconstruction procedures, which may restore vascularity possibly by stimulating the tissue biochemical milieu.

DCA is a neglected late complication of diabetes, often causing physical disability in performing skilled fine motor activities and thereby causing psychological and emotional disturbance. In developing countries, especially in rural setups with a lack of awareness, where most patients have poor adherence to treatment and follow-up, poor glycemic control, and nonadherence to recommended routine screening, physical examination for DCA can be a very useful simple screening test as a mirror for microvascular and macrovascular complications. Hence, if not done routinely or previously, workup for these complications may be advised as a mandatory measure in cases with DCA to detect complications like diabetic retinopathy at an early stage, and with appropriate interventions, further progression can be prevented. The main strategy in management of DCA includes physiotherapy, early stringent glycemic control, good patient education, and regular follow-up. Good centers for advanced surgical care and management can be developed for definitive management of deformity. DCA is an avoidable complication if type 1 diabetic patients are offered proper care with good glycemic control and regular follow-up. Planned long-term efforts can reduce the prevalence of this alarming and disabling complication, diabetic cheiroarthropathy.

## Learning Points

- DCA is a long-term complication of diabetes, causing physical and emotional disability.
- DCA is related to microvascular and macrovascular complications in varying proportions, most significantly diabetic retinopathy.
- The “Namaste” sign is a simple clinical tool to diagnose DCA.
- Screening for microvascular complications of diabetes may be emphasized, if not done previously, to patients presenting with DCA.
- Early and good glycemic control is the need of the hour in treating type 1 diabetic patients, to prevent DCA and other complications.

## Data Availability

Not applicable to this article as no datasets were generated or analyzed during the current study.

## Abbreviations

AGE: advanced glycation end-product
DCA: diabetic cheiroarthropathy
DLCO: diffusing capacity of the lungs for carbon monoxide
HRCT: high-resolution computed tomography
